# Evaluation of Expanded Mitral Regurgitation Grading in Patients Undergoing Transcatheter Edge-to-Edge Repair

**DOI:** 10.1016/j.shj.2024.100369

**Published:** 2024-10-26

**Authors:** Andrew Gustafson, O’Neil R. Mason, Blair Tilkens, Rishi Shrivastav, Kifah Hussain, Kevin Lin, Jyothy J. Puthumana, Akhil Narang

**Affiliations:** Division of Cardiology, Feinberg School of Medicine at Northwestern University, Chicago, Illinois, USA

**Keywords:** Mitral regurgitation, Effective regurgitant orifice area, Transcatheter edge-to-edge repair, Transesophageal echocardiography

## Abstract

**Background:**

An expanded tricuspid regurgitation scale has been shown to be incrementally useful in understanding the response to transcatheter therapies. A similar approach to mitral regurgitation (MR) has not been evaluated. The purpose of this study was to investigate how an expanded MR grading system that includes categories of massive and torrential would regrade patients undergoing transcatheter edge-to-edge repair (TEER) for MR and evaluate procedural outcomes.

**Methods:**

We retrospectively identified 142 consecutive patients with severe MR who underwent TEER. Transesophageal echocardiography was used to assess the quantitative severity of MR and reclassify regurgitation into severe, massive, and torrential grades. Similarly, residual MR was assessed postprocedurally.

**Results:**

In the expanded scale, 59% of patients were regraded as severe, 23% as massive, and 18% as torrential, with respective median effective regurgitant orifice area (cm^2^) of 0.45 [0.39, 0.50], 0.68 [0.65, 0.75], and 0.95 [0.85, 1.20]. Ninety-three percent of the entire cohort and 93% of severe, 94% of massive, and 96% of torrential patients, achieved moderate or less MR post-TEER (*p* = 0.850) with corresponding improvements in New York Heart Association Functional Classification and 12-item Kansas City Cardiomyopathy Questionnaire scores.

**Conclusions:**

An expanded grading system demonstrated that patients with massive and torrential MR still achieve adequate procedural success with reduction in regurgitation and improvement in health status. Further evaluation of how an expanded MR grading scale may be useful is warranted.

## Introduction

Recent studies have examined the impact of an expanded grading system in tricuspid regurgitation (TR) beyond severe to include massive and torrential.^1^, ^2^, ^3^ Some patients with TR may experience significantly more regurgitation by quantitative measurements, such as effective regurgitant orifice area (EROA), yet still be classified as “severe” under conventional valvular heart disease grading.^1^ Often, these patients may still be graded with “severe” TR after transcatheter intervention, despite quantitative TR improvement with corresponding improvement in quality of life and forward stroke volume.^1^ Patients with “massive” or “torrential” TR may also experience worsened mortality compared to patients with “severe” TR and may have worse outcomes after transcatheter tricuspid valve interventions.^2^^,^^3^ Similarly, worsened mortality has been demonstrated in patients with higher quantitative mitral regurgitation (MR) by EROA.^4^ The purpose of this study was to investigate how a similar expanded MR grading system that includes categories of massive and torrential would regrade patients undergoing transcatheter edge-to-edge repair (TEER) for MR and to further evaluate procedural and quality of life outcomes under an expanded MR grading system.

## Materials and Methods

### Study Population and Echocardiographic Acquisition

**Figure 1:**
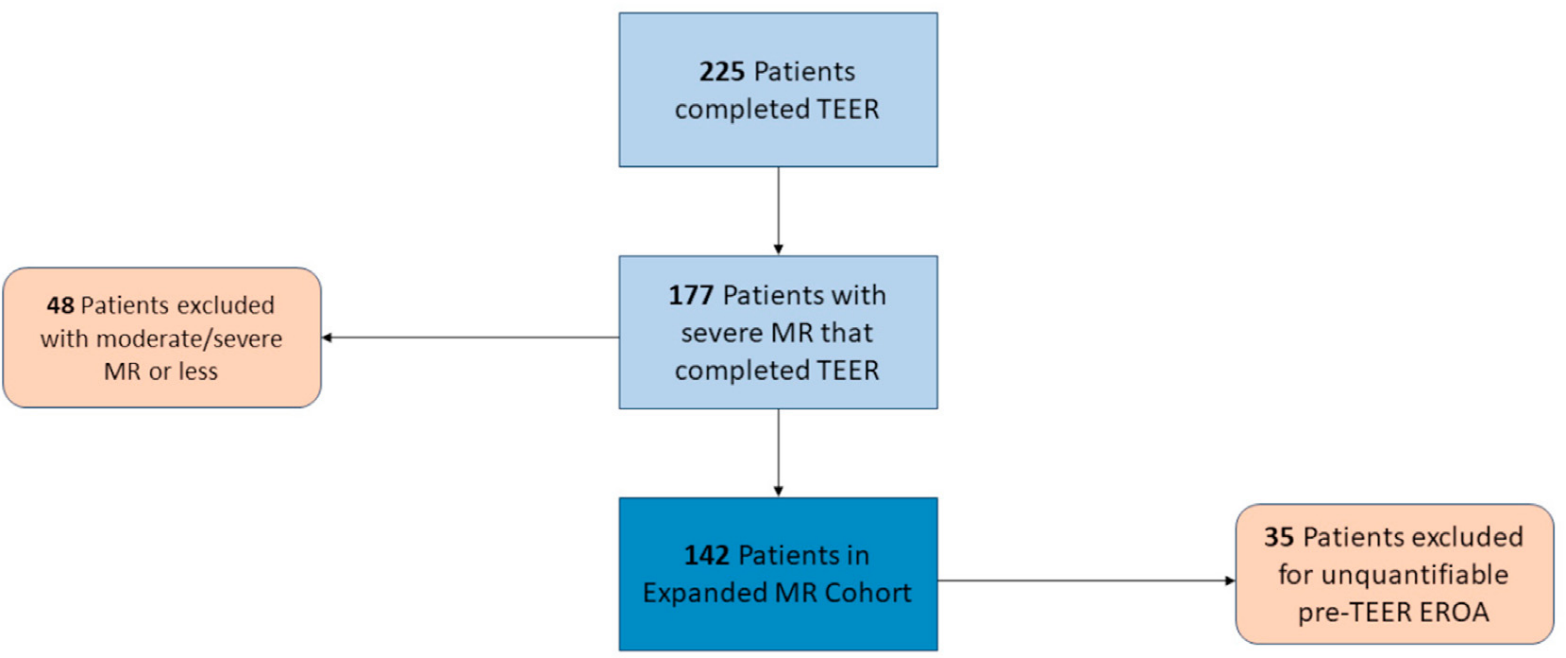
**Expanded MR cohort inclusion flowchart** Abbreviations: EROA, effective regurgitant orifice area; MR, mitral regurgitation; TEER, transcatheter edge-to-edge repair.

We retrospectively identified 225 patients who completed TEER for MR between July 2014 and March 2021. Forty-eight patients were excluded for preprocedural MR that was moderate-severe. A further 35 patients were excluded for unquantifiable EROA by flow convergence on retrospective review of transesophageal echocardiography images. Most of the unquantifiable studies had either a lack of quantitative imaging or inadequate proximal isovelocity surface areas (PISAs). Only patients with quantifiable pre-TEER EROA who completed TEER for severe MR were included in the final cohort (Figure 1). The preprocedural transesophageal echocardiography was reviewed by two echocardiographers independently to assess severity of MR via 2D EROA and regurgitant volume according to the American Society of Echocardiography/European Association of Cardiovascular Imaging standards.^5^^,^^6^ MR was regraded according to preprocedural EROA as follows: severe (0.40-0.59 cm^2^), massive (0.60-0.79 cm^2^), and torrential (≥0.80 cm^2^). Similarly, post-TEER, residual MR was assessed. Pre- and post-transthoracic echocardiograms were reviewed to assess periprocedural echocardiographic changes. Residual MR using both the conventional and expanded grading systems was subsequently compared. Post-TEER residual MR was assessed qualitatively when a PISA was not measurable (usually when MR was mild or less).

### Measurement of Health Status

Patient health status was assessed with Kansas City Cardiomyopathy Questionnaire-12 (KCCQ-12) scores pre-TEER and at 30 days postprocedurally.^7^, ^8^, ^9^ New York Heart Association (NYHA) Functional Classification was also recorded at the same encounters.

### Statistical Analysis

Baseline characteristics for the analytic cohort are presented as percentages for categorical variables and medians with interquartile ranges or means with SDs for continuous variables. The Society of Thoracic Surgeons Predicted Risk of Operative Mortality score for mitral valve replacement was used to estimate patient mortality risk.^10^ Baseline characteristics, echocardiographic parameters, and health status were compared by expanded MR grade. Continuous variables were compared using analysis of variance, and categorical variables were compared using χ^2^ tests. *P*-values <0.05 were considered statistically significant. The median KCCQ Summary Score (KCCQ-OS) at baseline and at 30 days post-TEER were compared across expanded MR grades using analysis of variance. Similarly, intraoperative and post-TEER echocardiographic parameters were compared across expanded MR grades using analysis of variance. The frequency of NYHA III/IV at baseline and at 30 days post-TEER was compared across expanded MR grades using χ^2^ tests. The post-TEER qualitative MR grade frequencies were also compared across expanded grades using χ^2^ tests.

## Results

### Study Sample

The median age of the entire cohort was 81 [72, 86] years with a median Society of Thoracic Surgeons score of 6% [4, 9]. Forty-six percent of the cohort was female; the etiology of MR was 77% primary, 14% secondary, and 9% mixed. Baseline patient characteristics are available in Table 1. Notably, higher frequencies of primary MR were observed with greater severity of MR (68% in severe, 82% in massive, 100% in torrential; *p* = 0.008) (Table 2).

**Table 1:** Patient characteristics by expanded MR

Variable	Severe		Massive		Torrential		Total		*P*-value
	N	Median (Q1, Q3)	N	Median (Q1, Q3)	N	Median (Q1, Q3)	N	Median (Q1, Q3)	
Age	84	80 (71, 85)	33	84 (74, 87)	25	84 (78, 87)	142	81 (72, 86)	0.169
STS score	70	6 (5, 9)	30	6 (4, 10)	22	7 (5, 9)	122	6 (4, 9)	0.863

Variable	N	Percentage	N	Percentage	N	Percentage	N	Percentage	*P*-value
Female	41	41/84 (49%)	13	13/33 (39%)	12	12/25 (48%)	66	66/142 (46%)	0.646
Diabetes	13	13/84 (15%)	7	7/33 (21%)	2	2/25 (8%)	22	22/142 (15%)	0.387
Dyslipidemia	46	46/84 (55%)	22	22/33 (67%)	17	17/25 (68%)	85	85/142 (60%)	0.327
Hypertension	58	58/84 (69%)	25	25/33 (76%)	16	16/25 (64%)	99	99/142 (70%)	0.614
CKD	21	21/84 (25%)	10	10/33 (30%)	5	5/25 (20%)	36	36/142 (25%)	0.575
CVD	12	12/84 (14%)	11	11/33 (33%)	3	3/25 (12%)	26	26/142 (18%)	**0.038**
Prior MI	16	16/84 (19%)	3	3/33 (9%)	2	2/25 (8%)	21	21/142 (15%)	0.226
Prior CABG	26	26/84 (31%)	5	5/33 (15%)	6	6/25 (24%)	37	37/142 (26%)	0.208
Prior PCI	21	21/84 (25%)	9	9/33 (27%)	5	5/25 (20%)	35	35/142 (25%)	0.811
Afib/Aflutter	55	55/84 (66%)	18	18/33 (55%)	11	11/25 (44%)	84	84/142 (59%)	0.132

*Notes.* Bolded values were found to be statistically significant differences between compared groups.

Abbreviations: Afib, atrial fibrillation; Aflutter, atrial flutter; CABG, coronary artery bypass graft; CKD, chronic kidney disease; CVD, cerebrovascular disease; MI, myocardial infarction; MR, mitral regurgitation; PCI, percutaneous coronary intervention; STS, Society of Thoracic Surgeons.

∗*P*-values were calculated by analysis of variance for continuous variables and *X*^*2*^ test for categorical variables.

**Table 2:** MR etiology by expanded grade

Variable	Severe		Massive		Torrential		Total		*P*-value
	N	Percentage	N	Percentage	N	Percentage	N	Percentage	
Primary	57	57/84 (68%)	27	27/33 (82%)	25	25/25 (100%)	109	109/142 (77%)	**0.008**
Mixed	18	18/84 (21%)	2	2/33 (6%)	0	0/25 (0%)	20	20/142 (14%)	
Secondary	9	9/84 (11%)	4	4/33 (12%)	0	0/25 (0%)	13	13/142 (9%)	

*Notes.* Bolded values were found to be statistically significant differences between compared groups.

Abbreviation: MR, mitral regurgitation.

∗*P*-values were calculated by *X*^*2*^ test for categorical variables.

### Periprocedural Echocardiographic Features and Echocardiographic Outcomes

In the expanded scale, 59% of patients were regraded as severe, 23% as massive, and 18% as torrential. At baseline, median EROA (cm^2^) was 0.45 [0.39, 0.50], 0.68 [0.65, 0.75], and 0.95 [0.85, 1.20], and median regurgitant volume (mL) was 72 [64, 85], 95 [83, 112], and 117 [97, 158] in the severe, massive, and torrential groups, respectively (Table 3). Severity of MR correlated with left ventricular ejection fraction (LVEF) (median LVEF 56% [45%, 62%] in severe, 60% [54%, 67%] in massive, and 69% [63%, 74%] in torrential; *p* ​< 0.001). Post-TEER, median EROA (cm^2^) was 0.14 [0.10, 0.25] in the entire cohort, 0.12 [0.08, 0.19] in the severe group, 0.24 [0.15, 0.29] in the massive group, and 0.14 [0.10, 0.17] in the torrential group among patients with quantifiable PISA (*p* = 0.025). Table 3 further summarizes transthoracic echocardiogram features post-TEER (median 28 days postimplantation).

**Table 3:** Periprocedural echocardiographic changes by expanded MR

Pre-TEER								Post-TEER						
Variable	Severe		Massive		Torrential		*P*-value	Severe		Massive		Torrential		*P*-value
	N	Median (Q1, Q3)	N	Median (Q1, Q3)	N	Median (Q1, Q3)		N	Median (Q1, Q3)	N	Median (Q1, Q3)	N	Median (Q1, Q3)	
EROA (cm^2^)	84	0.45 (0.39, 0.50)	33	0.68 (0.65, 0.75)	25	0.95 (0.85, 1.20)	***<*0.001**	28	0.12 (0.08, 0.19)	19	0.24 (0.15, 0.29)	11	0.14 (0.10, 0.17)	**0.025**
RVOL (mL)	60	72 (64, 85)	22	95 (83, 112)	18	117 (97, 158)	***<*0.001**	10	23 (19, 31)	8	35 (21, 47)	5	31 (20, 33)	0.596
LVEF (%)	64	56 (45, 62)	24	60 (54, 67)	10	69 (63, 74)	***<*0.001**	82	51 (36, 60)	32	58 (45, 63)	25	60 (55, 63)	**0.008**
LVEDV (mL)	53	118 (84, 145)	24	116 (97, 157)	10	114 (81, 150)	0.726	53	104 (71, 155)	32	114 (91, 172)	25	101 (88, 137)	0.267
LVESV (mL)	53	47 (35, 74)	24	46 (32, 67)	10	36 (25, 42)	0.142	53	50 (28, 71)	32	39 (38, 86)	25	42 (33, 63)	0.260
RVSP (mmHg)	55	46 (35, 54)	23	44 (39, 60)	11	47 (41, 64)	0.624	72	42 (33, 50)	32	48 (33, 56)	22	46 (40, 51)	0.218

*Note.* EROA and RVOL were from TEE pre- and post-TEER. RVOL, LVEF, LVEDV, LVESV, and RVSP were from pre- and post-TEER TTE (median 28 days post-TEER). Bolded values were found to be statistically significant differences between compared groups.

Abbreviations: EROA, effective regurgitant orifice area; LVEDV, left ventricular end diastolic volume; LVEF, left ventricular ejection fraction; LVESV, left ventricular end systolic volume; MR, mitral regurgitation; RVOL, regurgitant volume; RVSP, right ventricular systolic pressure; TEE, transesophageal echocardiography; TEER, transcatheter edge-to-edge repair; TTE, transthoracic echocardiogram.

∗*P*-values were calculated by analysis of variance for continuous variables and *X*^*2*^ test for categorical variables.

Pre-TEER MR grade was associated with post-TEER LVEF (median LVEF 51% [36%, 60%] in severe, 58% [45%, 63%] in massive, and 60% [55%, 63%] in torrential; *p* = 0.008). Median LVEF decreased in all expanded subgroups (56 to 51% in severe, 60 to 58% in massive, 69 to 60% in torrential). Median left ventricular end diastolic volume of the entire cohort decreased from 117 [88, 155] mL to 104 [88, 154] mL (11% reduction) at 1-month post-TEER with reduction in all expanded grades.

**Figure 2:**
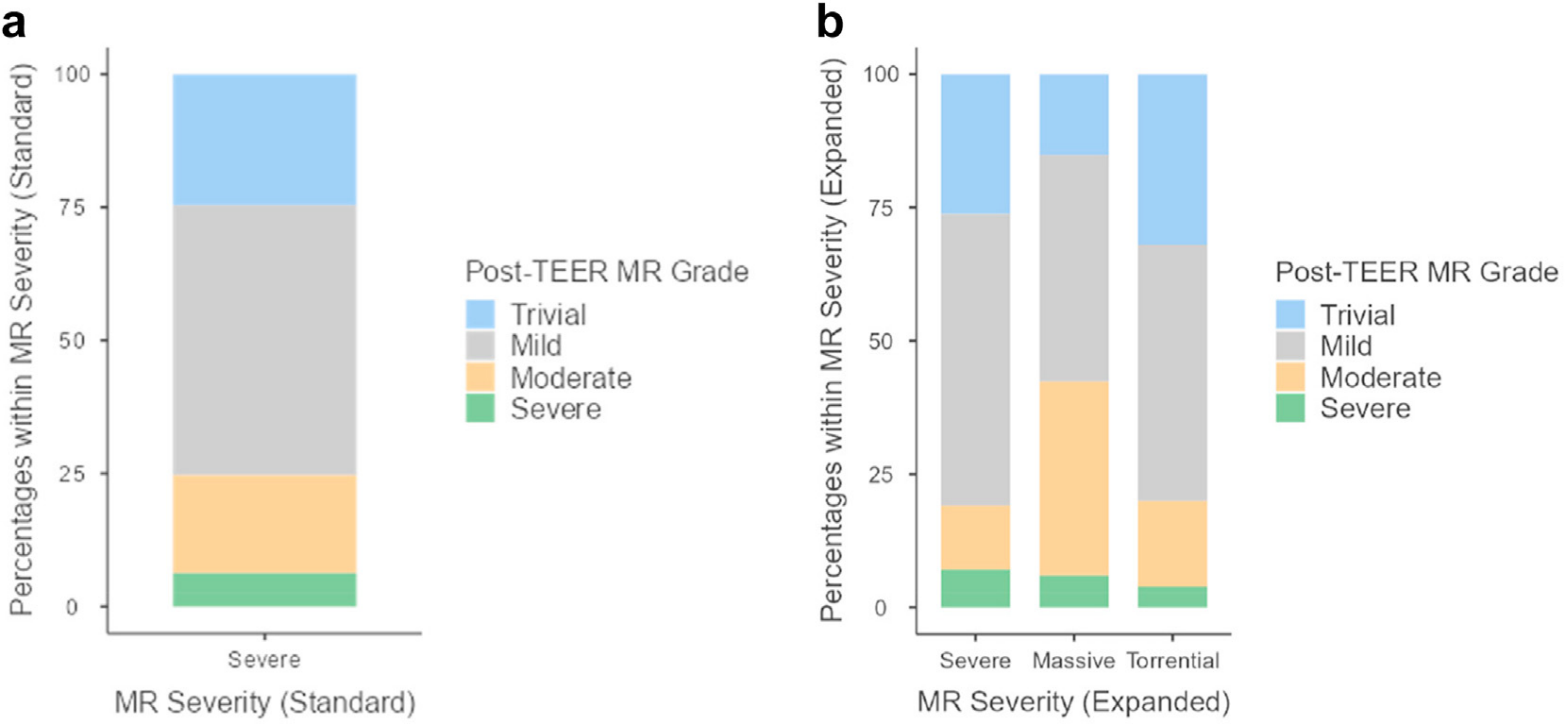
Qualitative post-TEER MR grade by (A) standard scale and (B) expanded scale Abbreviations: MR, mitral regurgitation; TEER, transcatheter edge-to-edge repair.

Post-TEER, 93% of the entire cohort achieved moderate or less MR; 93% of severe, 94% of massive, and 96% of torrential patients achieved moderate or less MR when studied by expanded grade (*p* = 0.850) (Figure 2). On subgroup analysis, 94%, 92%, and 95% with primary, mixed, and secondary MR, respectively, had moderate or less MR post-TEER, and 75% of the entire cohort achieved mild or less MR. In the overall cohort, patients experienced a mean decrease of 1.94 ​± ​0.83 grades when using the standard scale and 2.58 ​± ​1.12 grades when using the expanded scale (Table 4). In the expanded scale, MR grade decreased as follows: severe by 2.02 ​± ​0.79 grades, massive by 2.85 ​± ​0.87 grades, and torrential by 4.08 ​± ​0.81 grades (*p* < ​0.001). Mean transmitral gradient was 2.30 ​± ​1.45 mmHg pre-TEER and 3.56 ​± ​1.87 mmHg post-TEER. Pre-TEER, there were no statistical differences in mean gradients between expanded subgroups with gradients of 2.13 ​± ​1.17, 2.41 ​± ​1.79, and 2.67 ​± ​1.16 in the severe, massive, and torrential groups, respectively (*p* = 0.163). Likewise, post-TEER gradients were similar, with mean gradients of 3.46 ​± ​1.68, 3.95 ​± ​2.68, and 3.42 ​± ​1.26 in the severe, massive, and torrential groups, respectively (*p* = 0.898). A mean of 1.58 ​± ​0.58 MitraClips were used per case with the following on expanded subgroup comparison: severe 1.55 ​± ​0.59, massive 1.67 ​± ​0.60, and torrential 1.56 ​± ​0.51 (*p* = 0.587). Table 5 further describes the different MitraClips used in the cohort.

**Table 4:** Echocardiographic outcomes by expanded MR

Variable	Severe		Massive		Torrential		Total		*P*-value
	N	Mean, SD	N	Mean, SD	N	Mean, SD	N	Mean, SD	
Standard MR grades decreased	84	2.00 ​± ​0.82	33	1.67 ​± ​0.82	25	2.08 ​± ​0.81	142	1.94 ​± ​0.83	0.055
Expanded MR grades decreased	84	2.02 ​± ​0.79	33	2.85 ​± ​0.87	25	4.08 ​± ​0.81	142	2.58 ​± ​1.12	***<*0.001**
MitraClips per case	84	1.55 ​± ​0.59	33	1.67 ​± ​0.60	25	1.56 ​± ​0.51	142	1.58 ​± ​0.58	0.587

Variable	N	Percentage	N	Percentage	N	Percentage	N	Percentage	*P* value
Moderate or less MR post-TEER	78	78/84 (93%)	31	31/33 (94%)	24	24/25 (96%)	133	133/142 (93%)	0.850

*Notes.* Bolded values were found to be statistically significant differences between compared groups.

Abbreviations: MR, mitral regurgitation; TEER, transcatheter edge-to-edge repair.

∗*P*-values were calculated by analysis of variance for continuous variables and *X*^*2*^ test for categorical variables.

**Table 5:** Intraprocedural characteristics

Variable	N	Percentage
Number of Clips, Total	223	100%
NT Clips	168	75%
NTW Clips	0	0%
XT Clips	50	22%
XTW Clips	5	2%

### Health Status Outcomes

**Figure 3:**
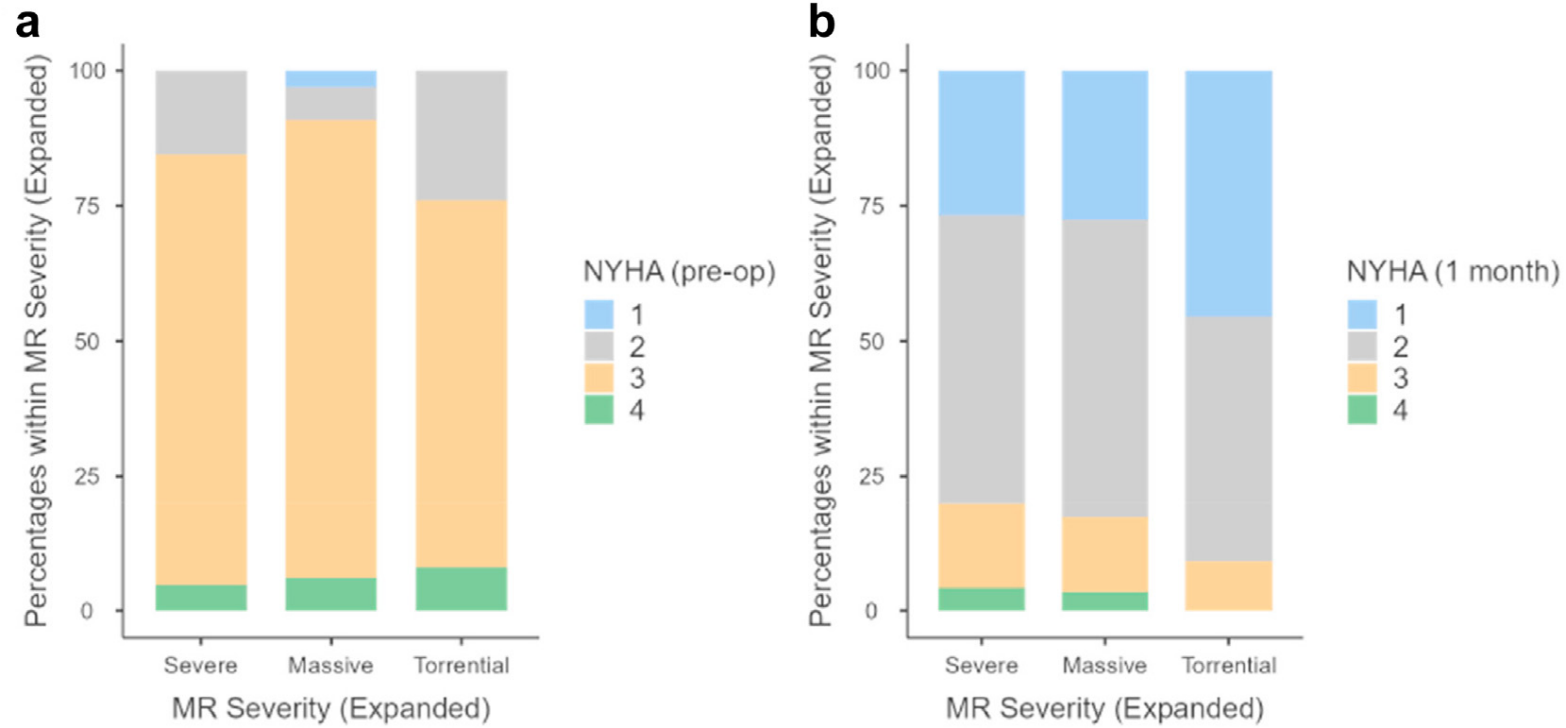
Comparison of NYHA class at (A) baseline and (B) 1 month by expanded MR grade Abbreviations: MR, mitral regurgitation; NYHA, New York Heart Association.

At baseline, the median KCCQ-OS in the entire cohort was 51 [35, 65]. One-month post-TEER, the median KCCQ-OS for the entire cohort had improved to 85 [66, 94] (Table 6). In subgroup analysis, median KCCQ improved in all expanded MR groups: severe (55 [35, 68] to 85 [65, 94]), massive (48 [29, 59] to 87 [63, 95]), and torrential (51 [35, 65] to 85 [66, 94]) without statistical significance between subgroups (*p* = 0.980). At baseline, 85% of the entire cohort, 85% of the severe, 91% of the massive, and 76% of the torrential subgroups had NYHA functional class III or IV symptoms (*p* = 0.300) (Figure 3). At 1-month post-TEER among patients with follow-up, 17% of the entire cohort as well as 20%, 17%, and 9% of the severe, massive, and torrential subgroups, respectively, had NYHA class III or IV symptoms (*p* = 0.514).

**Table 6:** Health status outcomes

Pre-TEER										Post-TEER								
Variable	Severe		Massive		Torrential		Total		*P*-value	Severe		Massive		Torrential		Total		*P*-value
	N	Median (Q1, Q3)	N	Median (Q1, Q3)	N	Median (Q1, Q3)	N	Median (Q1, Q3)		N	Median (Q1, Q3)	N	Median (Q1, Q3)	N	Median (Q1, Q3)	N	Median (Q1, Q3)	
KCCQ12 Summary	49	55 (35, 68)	25	48 (29, 59)	20	46 (35, 65)	94	51 (35, 65)	0.385	44	85 (65, 94)	27	87 (63, 95)	20	83 (72, 91)	91	85 (66, 94)	0.980
KCCQ12 Physical Limitation	49	63 (38, 75)	25	50 (42, 75)	20	56 (41, 75)	94	58 (39, 75)	0.613	42	88 (52, 100)	26	85 (59, 100)	20	85 (73, 94)	88	88 (58, 100)	0.979
KCCQ12 Symptom Frequency	49	56 (44, 67)	25	54 (44, 67)	20	59 (49, 71)	94	56 (45, 67)	0.805	45	83 (67, 96)	27	83 (71, 98)	20	84 (66, 94)	92	83 (67, 96)	0.867
KCCQ12 QOL	48	38 (13, 50)	25	25 (0, 50)	20	25 (13, 50)	93	38 (13, 50)	0.334	44	88 (63, 100)	27	75 (63, 100)	20	88 (63, 88)	91	88 (63, 100)	0.713
KCCQ12 Social Limitation	49	50 (25, 83)	24	50 (21, 69)	20	50 (33, 83)	93	50 (25, 75)	0.287	40	92 (73, 100)	26	88 (54, 100)	18	83 (77, 100)	84	91 (67, 100)	0.807

Abbreviations: KCCQ12, 12 item Kansas City Cardiomyopathy Questionnaire; QOL, quality of life; TEER, transcatheter edge-to-edge repair.

∗*P*-values were calculated by analysis of variance for continuous variables and *X*^*2*^ test for categorical variables.

## Discussion

The findings of our study of patients with significant MR who underwent TEER can be summarized as follows:

(1) Severe MR is a heterogenous disease process with many patients experiencing significantly greater degrees of quantitative regurgitation than other patients in the same conventional grade. An expanded MR grading scale may be useful in further characterizing the degree of valvular disease experienced by patients.
(2) A high percentage of patients can experience substantial reduction in MR with corresponding improvements in health status after TEER, regardless of MR etiology. Specifically, substantial MR reduction and improvement in health status can be achieved even in patients with the greatest quantitative MR by EROA.
(3) Further understanding of the utility of an expanded MR scale in describing outcomes following transcatheter therapies is needed.

Severe MR is likely a more nuanced disease process than current grading scales appreciate given the wide range of quantitative regurgitation observed in patients. Forty-one percent of our cohort had either massive or torrential MR under the expanded grading scale; many patients experienced significantly greater MR than others in the same conventional grade. For example, one patient’s MR was conventionally graded as severe despite having an EROA of 2.21 cm^2^. This may be particularly important prognostically, as higher EROA has been associated with higher all-cause mortality in primary and secondary MR.^4^ In subgroup analysis, 77% of the entire cohort had primary MR, and primary MR was more frequently observed with increasing severity of MR. This is in concordance with prior findings that described greater EROA values in primary compared to secondary MR.^4^^,^^11^ Other explanations include the tendency of 2D PISA to underestimate EROA with the crescentic shape of proximal convergence in secondary MR or the dynamic nature of MR throughout systole that may not be captured in a single frame.^11^^,^^12^ Investigating separate expanded MR grading scales for primary and secondary MR may be a direction for further investigation, particularly given the more significant impact on mortality with secondary MR.^4^

Even patients with the greatest quantitative MR experience substantial and reliable reduction in MR with improved quality of life after TEER. Expanded subgroups experienced comparable reduction of MR to moderate with at least 93% of patients in each subgroup achieving moderate or less MR post-TEER (*p* = 0.850). Operators used similar number of devices per case in each expanded subgroup, and patients experienced similar MR reduction regardless of MR etiology. The reduction in MR observed in all expanded groups is noteworthy given the association of residual MR with mortality and risk for heart failure hospitalization.^13^, ^14^, ^15^, ^16^

Recently, the TRILUMINATE trial demonstrated improved quality of life with transcatheter repair of TR, and the patients who had the largest reduction in TR experienced the greatest improvement in quality of life.^17^ The study of patients with greater initial quantitative MR and whether they derive incremental benefit from MR reduction may be a direction for future inquiry. In our cohort, all expanded subgroups experienced similar health status at 1-month post-TEER. Median 12-item KCCQ-OS scores improved by 28, 35, and 38 points in the severe, massive, and torrential subgroups, respectively, with improvements seen in every KCCQ category. While this was not statistically significant, the early signal of improved quality of life scores post-TEER with increasing baseline severity of MR is interesting. All expanded subgroups also had similar NYHA functional classifications at 1-month post-TEER, and only 9% of the torrential patients who responded had class III or IV symptoms. Correspondingly, median left ventricular end diastolic volume of the entire cohort decreased from 117 to 104 mL (11% reduction) at 1-month post-TEER—comparable to previous studies.^18^, ^19^, ^20^

The use of an expanded scale may be useful in describing the reduction in MR with intervention. Under the standard scale, patients experienced a mean decrease of 1.94 ​± ​grades—a qualitative assessment that may not reflect differing quantitative MR reduction between patients. In contrast, the application of an expanded scale emphasizes the incremental reduction of some patients—i.e., patients with torrential MR had a mean reduction of 4.08 ​± ​0.81 grades compared to the 2.02 ​± ​0.79 grade reduction observed in patients with severe MR. This may prove valuable given the limitations of post-TEER quantitative assessment with 2D methods and the current lack of data for the use of 3D methods in a variety of settings (i.e., outside of tertiary heart centers).^21^

### Limitations

This hypothesis-generating study has several limitations. First, it is a retrospective, observational study at one institution and is thus subject to bias and confounding. Second, post-TEER quantitative evaluation of MR was limited due to substantial MR reduction and the lack of suitable images for 3D quantification on retrospective review. Third, some patients were lost to follow-up and/or lacked data. Other considerations would be the incorporation of other TEER devices such as PASCAL and the use of 3D assessment for MR quantification, particularly for residual MR post-TEER.^22^^,^^23^

## Conclusions

Our overall findings indicate that severe MR is likely more nuanced than current grading systems appreciate and that an expanded scale may be useful in describing the substantial MR reduction that patients with the most significant MR experience post-TEER. Furthermore, patients across all expanded subgroups can experience similar post-TEER residual MR with similar functional health status. Further investigation into the long-term clinical benefits of transcatheter mitral valve repair according to an expanded grading scale is needed.

## Funding

A. Narang has funding from the Feis Family Foundation to report.

## Prior Presentation

An earlier version of this research was presented at the American Society of Echocardiography Scientific Sessions 2023 in Washington, D.C.

## Disclosure and Ethics Statement

A. Narang is a consultant for Abbott Laboratories, Edwards Lifesciences, and Bristol Meyers Squibb. J. Puthumana is a consultant for Abbott Laboratories. The other authors had no conflicts to declare. This study was approved by the institution IRB in accordance with best research guidelines and policies.
